# A dosimetric comparison of four treatment planning methods for high grade glioma

**DOI:** 10.1186/1748-717X-4-45

**Published:** 2009-10-21

**Authors:** Leor Zach, Bronwyn Stall, Holly Ning, John Ondos, Barbara Arora, Shankavaram Uma, Robert W Miller, Deborah Citrin, Kevin Camphausen

**Affiliations:** 1 Radiation Oncology Branch, National Cancer Institute, 10 Center Drive Building 10, CRC, Bethesda, MD, 20892 USA

## Abstract

**Background:**

High grade gliomas (HGG) are typically treated with a combination of surgery, radiotherapy and chemotherapy. Three dimensional (3D) conformal radiotherapy treatment planning is still the main stay of treatment for these patients. New treatment planning methods suggest better dose distributions and organ sparing but their clinical benefit is unclear. The purpose of the current study was to compare normal tissue sparing and tumor coverage using four different radiotherapy planning methods in patients with high grade glioma.

**Methods:**

Three dimensional conformal (3D), sequential boost IMRT, integrated boost (IB) IMRT and Tomotherapy (TOMO) treatment plans were generated for 20 high grade glioma patients. T1 and T2 MRI abnormalities were used to define GTV and CTV with 2 and 2.5 cm margins to define PTV1 and PTV2 respectively.

**Results:**

The mean dose to PTV2 but not to PTV1 was less then 95% of the prescribed dose with IB and IMRT plans. The mean doses to the optic chiasm and the ipsilateral globe were highest with 3D plans and least with IB plans. The mean dose to the contralateral globe was highest with TOMO plans. The mean of the integral dose (ID) to the brain was least with the IB plan and was lower with IMRT compared to 3D plans. The TOMO plans had the least mean D10 to the normal brain but higher mean D50 and D90 compared to IB and IMRT plans. The mean D10 and D50 but not D90 were significantly lower with the IMRT plans compared to the 3D plans.

**Conclusion:**

No single treatment planning method was found to be superior to all others and a personalized approach is advised for planning and treating high-grade glioma patients with radiotherapy. Integral dose did not reflect accurately the dose volume histogram (DVH) of the normal brain and may not be a good indicator of delayed radiation toxicity.

## Background

High grade gliomas (HGG) are the most prevalent primary malignant brain tumors in adults. These malignancies are typically treated with a combination of surgery, radiotherapy and chemotherapy. Three dimensional (3D) conformal radiotherapy treatment planning is still the main stay of treatment for these patients with treatment volume delineation based on Magnetic Resonance Images (MRI) fused to the patient's simulation computed tomography. New technologies for radiotherapy planning and treatment such as Intensity Modulated Radiotherapy (IMRT) and new treatment instruments such as Tomotherapy are becoming widely used. These provide better dose conformality, add certainty to dose delivery to the target volumes, and allow sparing of sensitive organs adjacent to the treatment field and/or escalation of the dose to the target volumes [1-7]. Although technically feasible, the clinical benefit of the use of these technologies in the treatment of HGG patients is unclear. Dose escalation in patients with HGG has thus far yielded disappointing results [1,2] and the use of advanced planning techniques to spare a presumably healthy tissue surrounding the primary lesions to reduce toxicity is of uncertain benefit [8]. Furthermore, the problematic quality assurance and reproducibility of some of these advanced treatment planning methods may compromise the ability to test them in a controlled randomized trial [3]. In the current study, we aimed to compare the dose distribution in target volumes as well as normal tissues with four treatment planning methods, done for the same patients with HGG. Our purpose for conducting this dosimetric comparison was to discover the benefits and drawbacks of each planning method. We also aimed to evaluate the ability of the calculated integral dose (ID) to reflect the actual dose distribution in the normal brain. A conformal three dimensional (3D) plan as well as a Linear Accelerator (LINAC, Varian Clinac-21EX equipped with the Millennium120 Multi Leaf Collimator) based sequential boost IMRT plan were generated. We generated a third LINAC based plan that was also an IMRT plan but was prescribed as an Integrated Boost (IB) plan. Tomotherapy (TOMO) plans were also generated for each patient using IB dose prescription.

## Methods

Twenty adult patients with high grade glioma, previously treated with conventional 3D conformal radiotherapy at the Radiation Oncology Branch of the National Cancer Institute during the period 2004-2008, were included in this study. Available treatment planning simulation CT images and diagnostic contrast enhanced pre-operative T1 and T2 MR Images were mandatory for the patients to be included.

The contrast enhanced MR images were fused to the simulation CT images using the Eclipse planning system (Varian Medical Systems, Palo Alto, CA). For each patient, a Gross Tumor Volume (GTV) and a Clinical Target Volume (CTV) [9] were contoured using the contrast enhanced T1 and the T2 MRI abnormalities, respectively. A 2-cm margin to the CTV was used to define the Planning Target Volume 1 (PTV1 [9]), and a 2.5-cm margin to the GTV was used to define PTV2. Areas of the PTV1 and PTV2 that were outside the skull were trimmed with 0.5 cm inner margin to the body contour. The globes and the optic chiasm were contoured and were designated as organs at risk during the treatment planning. The brain stem, subventricular zones (SVZ) and the normal brain (the volume of brain that was left after excluding the PTV1 and PTV2 volumes using the software Boolean operators) were also contoured for toxicity evaluation, but were not taken into consideration during treatment planning as organs at risk. The SVZs, which are believed to harbor the brain progenitor cells [10], were contoured as previously described [11]. Briefly, the lateral ventricles were contoured in both sides of the brain. The lateral edges of the ventricles were marked using a brush tool with the width of 0.5 cm. Treatment volumes and normal structures were contoured by a single physician and verified by a second physician.

For each patient, four treatment plans were generated. Three LINAC based treatment plans included a 3D plan, an IMRT plan and an IB IMRT plan. These were done for Varian Clinac-21EX beams. This machine is equipped with the Millennium120 multi leaf collimator (MLC). The leaf width for the central 40 pairs is 5 mm and for the outer 20 pairs is 1 cm. The simulation CT images and associated contours were then transferred from the Eclipse treatment planning software to the Tomotherapy treatment planning software (TomoTherapy Inc., Madison, WI) using the DICOM-RT protocol to generate the fourth treatment plan with the Tomotherapy treatment planning station. The optimal beam arrangement that delivered optimal tumor coverage and normal tissue sparing was selected after comparisons of various beam arrangements. Dose constrains and priorities were modified as needed in the IMRT, IB and Tomotherapy algorithms during the optimization process. The 3D LINAC plans typically included 3-5 treatment fields to conform the dose for each target volume and the IMRT plans included typically 4-5 non co-planar treatment fields. If possible the dose to contra-lateral brain was limited, when this did not compromise the dose to critical structures. The beams were chosen accordingly. The IMRT and IB plans were identical in field arrangement and differed only by the dose prescription parameters. The helical Tomotherapy parameters definitions were 1 cm for the field size (slice thickness) and 0.2-0.3 for pitch (the ratio of the distance the couch travels to the field width per one full rotation of the gantry). The Planning Modulation Factor (the ratio between the longest time a leaf is opened to the mean leaf opening time) was 2.00 and the mean actual modulation factor was 1.71 (range 1.22-1.96). The Plan Calculation Grid (image resolution for dose calculations) was typically 0.274*0.274 cm (range 0.196*0.196 cm to 0.424*0.424 cm).

**Figure 1:**
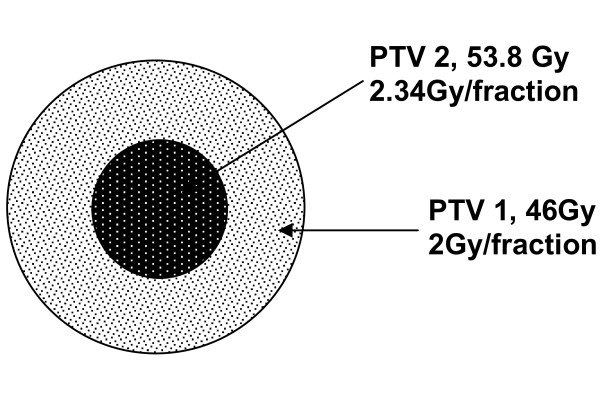
**Schematic illustration of the Integrated Boost target volumes and dose prescription**. Abbreviation: PTV1 - Planning Treatment Volume 1 (corresponds with T2 MR Image abnormality with 2 cm margins), PTV2 - Planning Treatment Volume 2 (corresponds with T1+ contrast MR Image abnormality with 2.5 cm margins).

Dose calculations for all plans were based on photon beams with maximal energy of 6-15 MV. The 3D and IMRT plans included two sequential plans each, using the PTV1 and PTV2 (boost) as the target volumes, with 46 Gy in 23 fractions and 14 Gy in 7 fractions, respectively, as the prescribed doses. Both the LINAC based IB plan and the TOMO plan were prescribed as integrated boost plans as previously described [4]. Briefly, the integrated boost included 23 fractions in a single plan, with a differential dose prescription to the target volumes. A total dose of 46 Gy in 2 Gy fractions was prescribed to PTV1 and a total dose of 53.8 Gy in 2.34 Gy fractions was prescribed for PTV2. The PTV2 total dose was calculated as the bioequivalent dose of 30 fractions of 2 Gy given in 23 fractions according to the linear quadratic model with a α/β ratio of 3. The integrated boost concept is illustrated in figure 1. Acceptable inhomogeneity was defined as 5% above and 7% below the prescribed dose inside the target volumes. An inhomogeneity coefficient (IC) of the dose in the target volumes was calculated using the formula (Dmax-Dmin)/Dmean as previously described [12]. The closer the IC to zero, the more homogenous the plan was considered.

The maximal dose allowed to the optic chiasm was 54 Gy, in the 3D and IMRT plans and 51.5 Gy in the IB and TOMO plans. An effort was made to keep the dose to the globes below 5 Gy. Chiasm but not globes constrains had higher priority than the target volume inhomogeneities in the IMRT, IB and TOMO optimization algorithms when there was an overlap of the structures. The brain stem, the subventricular zones and the normal brain did not have dose constrains during treatment planning. Bioequivalent dose calculations were used to allow the comparison of the IB and TOMO plans to the 3D and IMRT plans.

**Figure 2:**
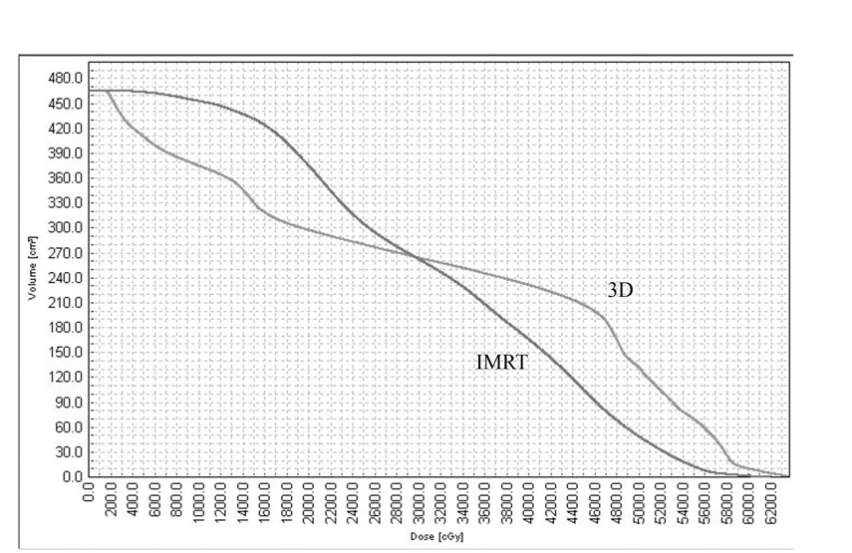
**A sample Dose Volume Histograms (DVH) of normal brain dose with two different plans**. Although the Integral Dose to the brain according to these to DVH's is the same (15.4 Gyxcm^3 ^× 1000), it is obvious that these histograms are different in both high and low dose areas. The dose received by 10% of the brain volume (D10) and 90% of the brain volume (D90) can describe more accurately such a difference. Abbreviation: NA-not applicable. SD-Standard Deviation.

Two methods were used to compare the dose distribution in the normal brain. First, the Integral Dose (ID) was calculated as previously described. Briefly, the volume of the normal brain was multiplied by the mean dose to the brain [12,13]. Since different dose volume histogram (DVH) curves can generate the same ID value (figure 2) we decided to compare three points on the DVH of the normal brain in each plan. D10, D50 and D90 represent the dose received by 10%, 50% and 90% of the normal brain volume, respectively

The mean percent volume coverage of the target volumes as well as the mean of the maximal dose to normal organs were calculated for each plan. The means of the IC, the normal brain ID and D10, D50 and D90 to the brain were also calculated. Since all these values relate to the same volumes, there was no need to normalize them for the purpose of this comparison.

An Excel based (Microsoft Office) two-tailed paired student T test was used to determine if there was a statistically significant difference between the means of the above values accomplished by each treatment planning method. A statistically significant difference was defined when the T test resulted in a p value of < 0.05.

## Results

### Patients' characteristics

A total of 20 patients were included in our study, 11 males and 9 females with a mean age of 54 y (range 37-71). Nineteen patients had a pathological diagnosis of Glioblastoma Multiforme (GBM, World Health Organization grade IV) and one had Anaplastic Astrocytoma (AA, World Health Organization grade III).

### Target volumes' coverage

The mean PTV1 and PTV2 volumes were 452 cm^3 ^(range 276-1074 cm^3^) and 300 cm^3 ^(range 137-567 cm^3^) respectively. A mean of >98% (range 92-100%) of the PTV1 received 100% of the prescribed dose in all planning methods. A mean of 95.5% and 95.7% of the PTV2 received 100% of the prescribed dose with TOMO and IMRT plans respectively. A mean of 94% and 92% of the PTV2 received 100% of the prescribed dose with IB and 3D treatment plans, respectively. The mean IB and 3D plans PTV2 coverage was significantly inferior then the IMRT and TOMO plans (p < 0.02, for all comparisons).

**Figure 3:**
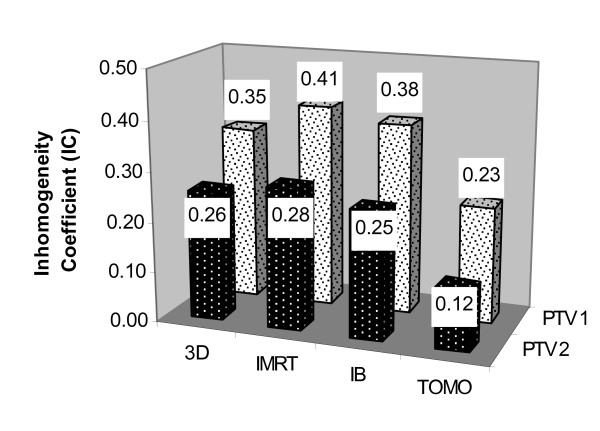
**The mean Inhomogeneity Coefficient (IC) achieved by the different planning methods**. The mean of the Inhomogeneity Coefficient is a measure of dose inhomogeneity in the target volumes. The closer the IC to zero, the more homogenous the dose is.

The mean Inhomogeneity Coefficient (IC) was significantly lower (better) with the TOMO plans, compared to all other plans for both PTV1 and PTV2 (p < 0.0003 for all comparisons) (figure 3). The mean IC of the IMRT plans was significantly higher (worse) than the mean IC of the 3D plans regarding PTV 1 (p < 0.02) and higher (worse) then the mean IC of the IB plans regarding PTV2 (p < 0.03) (figure 3). No significant difference was found between the means of the IC of the 3D and IB plans.

### Normal tissue sparing

**Figure 4:**
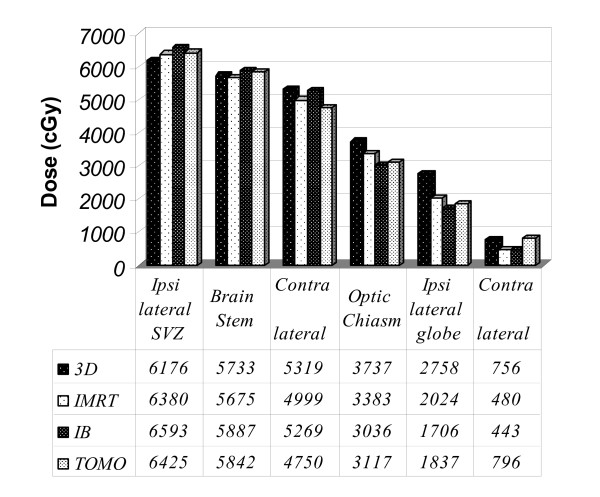
**The mean maximal dose (cGy) in normal tissues found with each treatment planning method**. Abbreviations: SVZ- sub ventricular zone, 3D- conformal three dimensional, IMRT-Intensity Modulated Radio Therapy, IB-Integrated Boost, TOMO - Tomo Therapy.

The mean of the maximal dose to the normal structures with the various treatment plans was used as a surrogate to normal tissue sparing [14] (figure 4). The optic chiasm, which was designated as organ at risk during treatment planning, was better spared with all other planning methods compared to the 3D plan (p < 0.05 for all comparisons). The IB plan (p < 0.00002) but not the TOMO plan (p > 0.5) spared the optic chiasm significantly better then the IMRT plan. All plans met the pre defined dose constrains for the optic chiasm.

The TOMO and IB plans spared the ipsilateral globe significantly better then the 3D plans (p < 0.002, p < 0.0008 respectively) but only the IB plan spared that globe significantly better then the IMRT plan (p < 0.005). No significant difference was found between the IB and the TOMO sparing of these organs (p > 0.3).

The contralateral globe in contrast, was not spared with the TOMO plan which had the highest mean maximal dose compared to all other plans (p value < 0.0001 compared to the IMRT and IB plans and p > 0.05 compared to the 3D plan). No other significant difference was found between the plans in respect to the ability to spare the contralateral globe. Although the *mean *dose of the IMRT and IB plans to the contralateral globe was lower then the 3D plan, a careful evaluation of the dose to that globe in some individual cases was lowest with the 3D plan compared to all others (data not shown).

The mean maximal dose to the ipsilateral subventricular zone (SVZ) was the highest with the IB treatment planning followed by TOMO, IMRT and 3D (p < 0.05 for all comparisons except IMRT vs. TOMO plans). The contralateral SVZ was best spared using the IMRT (p < 0.05 compared to every other planning method). No significant difference was found between the IB, 3D and TOMO plans in this respect.

The mean maximal dose to the brain stem with IMRT plan was significantly lower than with IB and TOMO (p < 0.008 for both comparisons) but not the 3D plan (p > 0.6).

**Figure 5:**
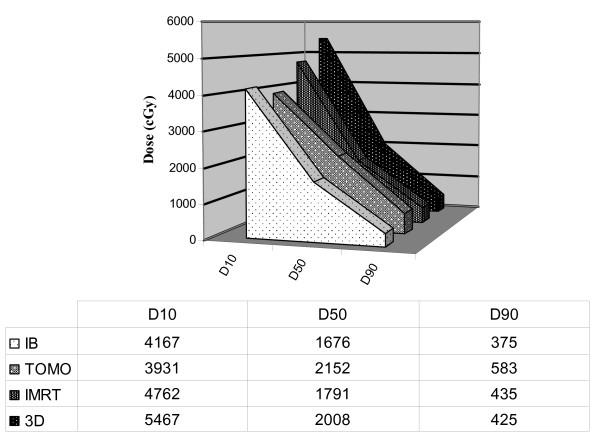
**The mean D10, D50, and D90 found with each treatment planning method**. The mean dose to 10% (D10), 50% (D50) and 90% (D90) of the normal brain volume (after the PTV1 and PTV2 volumes were excluded using the software Boolean Operators) with each treatment planning method.

The mean of the integral dose (ID) to the brain was significantly lower (range 10-17.5%) with the IB plan compared to all other plans (p < 0.006) (table 1). No significant difference in the ID to the brain was found among the other plans. A different pattern was noted when the D10, 50 and 90 were extracted out of the DVHs of the various plans and compared. The mean dose to 10 percent of the normal brain was consistently lowest with the TOMO plans followed by the IB, IMRT and 3D plans. Conversely, the D50 and D90 values were significantly higher with the TOMO plans compared to both IB and IMRT plans and the D50 but not the D90 was significantly lower in the IB plan compared to the IMRT and 3D plans. Interestingly, IMRT D10 and D50 were significantly lower than the 3D doses, but no significant difference was found between these plans regarding D90 (figure 5).

**Table 1:** The mean Integral Dose (ID) to the brain with each treatment planning method.

		**% Difference (p value)**		
**Integral Dose ± SD (Gy × cm^3 ^× 1000)**	**Plan**	***3D***	***IMRT***	***IB***
22.8 ± 7.2	***3D***	NA		
21.1 ± 3.7	***IMRT***	7.5 (0.2)	NA	
18.8 ± 3.1	***IB***	17.5 (0.006)*	10.9 (8.7E-09)*	NA
21 ± 2.3	***TOMO***	7.8 (0.3)	0.3 (0.6)	10.6 (0.003)*

* Statistically significant

## Discussion

In this study, we compared the delivery of radiation dose to the target volumes and the adjacent normal structures in high-grade glioma patients by using four treatment planning methods. This kind of comparison harbors numerous biases due to the use of different planning software, different optimization algorithms and different dose prescriptions. The use of mean values to compare these plans harbors another potential bias since it fails to reflect a better dose profile offered for individual patients by a specific planning method (e.g. target volume dose goals). Furthermore, a lower dose to a normal tissue is an important goal in treatment planning (e.g. SVZ or normal brain), but does not necessarily give an advantage if the tolerance of that tissue is not met (e.g. the optic chiasm).

A qualitative comparison of the various plans is summarized in table 2. According to our results, there is no single treatment planning method that is superior to all others in all aspects compared.

**Table 2:** A qualitative comparison of the four treatment planning methods.

	**3D**	**IMRT**	**IB**	**TOMO**
**Target Volumes**				
*PTV1 coverage*	**+**	**+**	**+**	**+**
*PTV2 coverage*	**-**	**+**	**-**	**+**
*Inhomogeneity Coefficient*		**-**		**+**
**Normal Tissues Sparing**				
*Optic Chiasm*	**-**		**+**	
*Ipsilateral Globe*	**-**		**+**	**+**
*Contralateral Globe*	**+**			**-**
*Ipsilateral SVZ*	**+**		**-**	
*Contralateral SVZ*		**+**		
*Brainstem*		**+**	**-**	**-**
*Normal Brain ID*			**+**	
*Normal Brain D10*	**-**			**+**
*Normal Brain D50*	**-**		**+**	**-**
*Normal Brain D90*				**-**

+ Significant advantage - Significant disadvantage

### Target volumes' coverage

Sequential boost plans assume 100% dose coverage to the boost volume by the initial part of the treatment. In glioma patients, were this is not always the case (PTV2 is not a geometrical cone down of PTV1); cold spots might be noticed within the boost volume. Non standard target volumes [4,5] can overcome this problem but these were not tested prospectively [15].

More treatment fields may suggest some advantage in the PTV2 dose coverage (IMRT plan was better then the 3D plan).

Surprisingly, the PTV2 dose coverage with the IB plan was worse then the IMRT plan despite the use of the same beam arrangement and dose constrains. As previously reported [4], there is a trade off between the target volume coverage and the homogeneity of the dose (which is supported by our IC results). Prescribing the IB plans to a lower isodose line improved the coverage but compromised the homogeneity of the dose in the target volume (data not shown). The beam weighing algorithm used to produce a plan sum in a sequential boost (IMRT) compared to an integrated boost (IB) gives another explanation to the different PTV2 coverage [4].

The TOMO plan did not result in an inferior PTV2 coverage and achieved the best IC as well since it uses infinite number of fields by definition.

### Normal tissue sparing

The TOMO plans were able to spare best small organs which usually lie close to the target volumes and were highly prioritized (e.g. the chiasm and ipsilateral globe) but the advantage was relatively small compared to the IMRT and IB plans. The ability of the TOMO to spare these organs in patients with tumors located distant from them is questionable since blocking both the entering and exit doses to a distant volume without compromising the target volume coverage, is unlikely with the helical beam arrangement.

No benefit and even a potential disadvantage was found with the IB and TOMO plans for structures with larger volumes that often overlap with the target volumes, such as the SVZ and the brain stem (due to a higher dose per fraction translated into higher total BED in these areas). We chose not to give dose constrains to these structures and compared their dose distribution after the plans were generated. The SVZs (believed to harbor the normal brain progenitor cells [10]) viability is correlated with late radiation toxicity [11,16,17] on one hand justifying an attempt to lower their dose [11,18] but are also suspected as the source of cancerous stem cells in primary brain tumors, associated with the tumor ability to resist radiation treatment and recur[19-21]. Higher radiosensitivity of the brain stem compared to other parts of the brain is not reported and its tolerance to fractionated radiotherapy appears to be a function of the volume receiving high dose rather than the maximum point dose [22].

Integral Dose (ID) is the value usually used to compare the dose received by healthy tissues outside the target volumes [12-14]. We added an analysis of three points in the DVH curve (D10, D50 and D90) of the normal brain to evaluate the validity of the ID as a tool for this kind of comparisons since different DVH curves may result in the same ID. The TOMO plans, with the high conformality and a rapid fall off of the dose around the target volumes had the lowest D10 to the normal brain (only a small volume around the target volume received a high dose). At the same time, larger volumes of normal brain received irradiation at all (high D50 and D90). This significant dose of irradiation received by the normal brain was not demonstrated when the ID values were compared. Along with the expected longer survival of HGG patients in the future these differences may translate to toxicity. Lower ID values with IMRT compared to 3D plans were used to justify its use for HGG patients [14]. Our mean ID failed to indicate such a difference but it is in line with the lower D10 and D50 for the IMRT plans we found. We suggest caution in the use and interpretation of ID for treatment plans comparisons due to its failure to predict differences in DVH curves which might have significant implications in the future.

## Conclusion

Our data suggest a distinctive approach in the use of new treatment planning tools. Further investigation is indicated to better choose the correct tool for each patient. Larger series might suggest a decision algorithm according to the patient's tumor size, location and prognosis. Long follow up periods with HGG patients are becoming a widespread phenomenon, and may allow better understanding of the effect of the different DVH curves of the normal brain and the SVZ. A close follow up on patient's toxicity profiles and correlations with a specific treatment planning method are indicated. The use of imprecise and insensitive tools like ID to compare potential toxicity due to large irradiated volumes should be discouraged and better tools should be developed.

## Competing interests

The authors declare that they have no competing interests.

## Authors' contributions

LZ carried out the contouring, and participated in the study design, coordination, treatment planning and writing of the manuscript. BS participated in contouring and helped revising the draft manuscript. HN carried out the treatment planning. JO carried out MRI fusions and participated in treatment planning. BA participated in treatment planning. US participated in the statistical analysis of the results and helped revising the draft manuscript. RWM participated in treatment planning. DC participated in the data analysis and helped revising the draft manuscript. KC conceived of the study, and participated in its design and coordination and helped to draft the manuscript. All authors read and approved the final manuscript.
